# Mitochondria in Health and Diseases

**DOI:** 10.3390/cells9051177

**Published:** 2020-05-09

**Authors:** Sabzali Javadov, Andrey V. Kozlov, Amadou K. S. Camara

**Affiliations:** 1 Department of Physiology, School of Medicine, University of Puerto Rico, San Juan, PR 00936-5067, USA; 2 Ludwig Boltzmann Institute for Experimental and Clinical Traumatology, 1200 Vienna, Austria; 3 Department of Anesthesiology, Medical College of Wisconsin, Milwaukee, WI 53226, USA; 4 Department of Physiology, Medical College of Wisconsin, Milwaukee, WI 53226, USA; 5 Cancer Center, Medical College of Wisconsin, Milwaukee, WI 53226, USA

**Keywords:** mitochondria, energy metabolism, signaling pathways, ion homeostasis, human diseases

## Abstract

Mitochondria are subcellular organelles evolved by endosymbiosis of bacteria with eukaryotic cells characteristics. They are the main source of ATP in the cell and play a pivotal role in cell life and cell death. Mitochondria are engaged in the pathogenesis of human diseases and aging directly or indirectly through a broad range of signaling pathways. However, despite an increased interest in mitochondria over the past decades, the mechanisms of mitochondria-mediated cell/organ dysfunction in response to pathological stimuli remain unknown. The Special Issue, “Mitochondria in Health and Diseases,” organized by *Cells* includes 24 review and original articles that highlight the latest achievements in elucidating the role of mitochondria under physiological (healthy) conditions and, in various cell/animal models of human diseases and, in patients. Altogether, the Special Issue summarizes and discusses different aspects of mitochondrial metabolism and function that open new avenues in understanding mitochondrial biology.

## 1. Introduction

**Figure 1:**
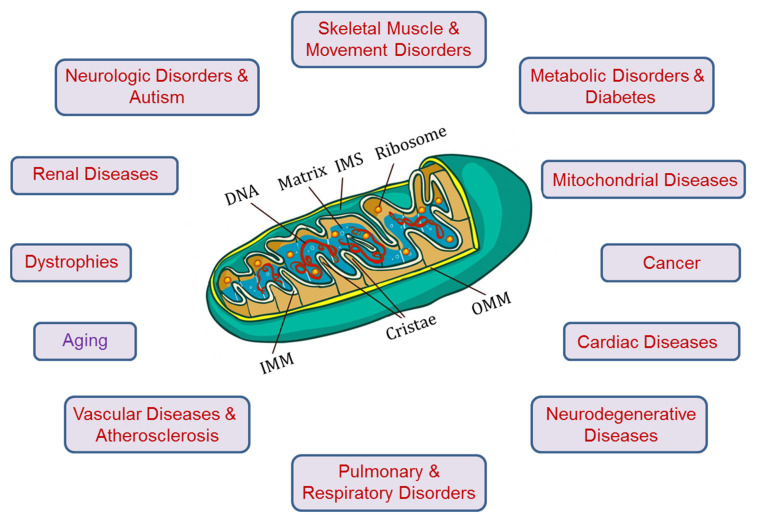
Mitochondria are involved in the pathogenesis of human diseases, and aging. IMM, inner mitochondrial membrane; IMS, intermembrane space; OMM, outer mitochondrial membrane.

Mitochondria have been recognized as the “power plants” that provide over 90% of ATP required for cell metabolism. Also, they are engaged in other aspects of cell metabolism and function and participate in the regulation of ion homeostasis, cell growth, redox status, cell signaling, and, thus, play a pivotal role in both cell survival and cell death mechanisms. Due to their central role in cell life and death, mitochondria are also involved in the pathogenesis and progression of numerous human diseases, including, among others, cancer, neurodegenerative and cardiovascular disorders, diabetes, traumatic brain injury, and inflammation (Figure 1). Mitochondria involvement in these diseases has been attributed to the pivotal role the organelle plays in the sequelae of events that culminate in cell death through various programmed (apoptosis, necroptosis, pyroptosis, ferroptosis, and autophagy) and non-programmed (necrosis) cell death mechanisms. A growing body of evidence on the important role of mitochondria under physiological conditions and human diseases is associated with increased number of biomedical studies in mitochondrial research. Since 2010, the number of mitochondria-related publications has exceeded other organelles including the nucleus, endo(sarco)plasmic reticulum, and the Golgi apparatus [1].

**Figure 2:**
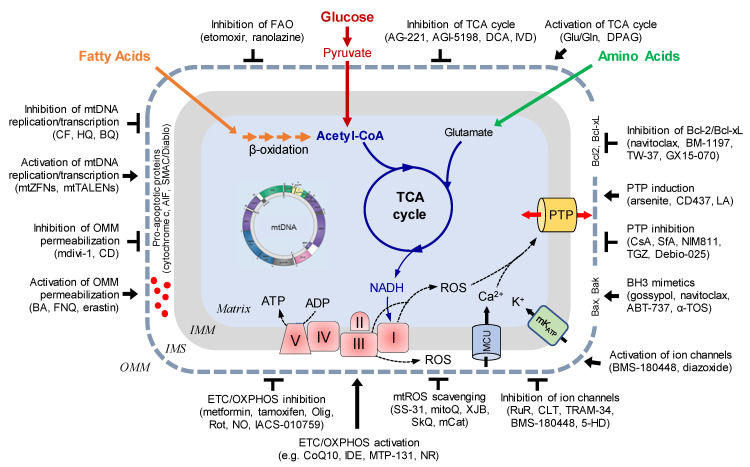
Mitochondria are promising therapeutic target for human diseases. *Abbreviations:* AIF, apoptosis-inducing factor; BA, betulinic acid; BQ, p-benzoquinone, mCAT, mitochondrial-targeted catalase; CD, chlorodiazepam; CF, ciprofloxacin; CLT, clotrimazole; CsA, cyclosporin A; DCA, dichloroacetate; DPAG, dipyruvyl-acetyl-glycerol; FAO, fatty acid oxidation; FNQ furanonaphthoquinone; 5-HD, 5-hydroxydecanoate; HQ, hydroquinone; Glu/Gln glutmate/glutamine; IDE, idebenone; IMM, inner mitochondrial membrane; IMS, intermembrane space; IVD, ivosidenib; mK_ATP_, mitochondrial ATP-sensitive potassium channel; LA, lonidamine; MCU, mitochondrial calcium uniporter; NR, nicotinamide riboside; Olig, oligomycin, OMM, outer mitochondrial membrane; PTP, permeability transition pore; Rot, rotenone; RuR, ruthenium red; SfA, sanglifehrin A; mtTALENs, mitochondrially targeted transcription activator like effectors (TALE) fused with a Fok1 nuclease; α-TOS, α-tocopheryl succinate; TGZ, troglitazone; mtZFNs mitochondrially targeted zinc finger nucleases. *The names of representative compounds are shown in brackets.*

Increased attention to mitochondria in recent decades stimulated preclinical studies on various cell and animal models to elucidate mitochondria as a therapeutic target for the treatment of a broad spectrum of human diseases. A large number of preclinical studies demonstrated beneficial effects of various pharmacological agents targeting mitochondrial ion channels, electron transfer chain (ETC), oxidative phosphorylation (OXPHOS), tricarboxylic acid (TCA) cycle, reactive oxygen species (ROS) production, permeability transition pore, DNA, membrane integrity, and apoptotic proteins, among others (Figure 2). However, despite an increased number of clinical trials conducted during recent decades, no single mitochondria-targeted compound has been approved by the U.S. FDA (Food and Drug Administration) so far that could be clinically applicable. Failure of the clinical trials can be explained by the fact that precise mechanisms whereby mitochondria are involved in the regulation of basic physiological functions, as well as their role in the cell under pathophysiological conditions, remain unknown. Furthemore, the mechanisms of interactions between mitochondria and other subcellular compartments and organelles such as endo(sarco)plasmic reticulum, nucleus, and lysosomes remain to be elucidated. Lack of in-depth knowledge of the regulatory mechanisms of mitochondrial metabolism and function as well as interplay between the factors that transform the organelle from the pro-suvival player to the pro-death contributor has hindered the development of new mitochondria-targeted pharmacological and conditional approaches for the treatment of human diseases.

To further improve our understanding of mitochondria, *Cells* organized the Special Issue entitled “Mitochondria in Health and Diseases” to highlight the latest achievements in elucidating mitochondrial metabolism and function under physiological and pathological conditions. The Special Issue published 24 articles consisting of 5 review articles and 19 original articles that cover a broad spectrum of mitochondrial research. 

## 2. Mitochondria in Health

This section of the Editorial focuses on the role of mitochondria in maintaining normal and healthy physiology. In this endeavor, 13 articles (10 original research and 3 review articles) elucidating different aspects of mitochondria in maintaining health in the organism have been published. These studies can be grouped in the following four sections: (1) *Mitochondrial homeostasis*, (2) *Mitochondrial and cellular metabolism*, (3) *Crosstalk between mitochondria and other subcellular compartments*, and (4) *Mitochondrial ion channels*. The review articles highlight (a) the significance of mitochondria crosstalk with cytoskeletal proteins key in normal mitochondrial and cellular physiology, (b) mitochondrial gene regulation in different cellular contexts, and the importance of emerging aspects of mitochondrial transcripts and gene regulation in human health and disease, and (c) the role of telomeres and telomerase in cardiac aging. Altogether, these seminal articles provide a broad spectrum of new and unique perspectives in our understanding of the role of mitochondria in health and disease. 

*Mitochondrial homeostasis*: To preserve themself and the host cell, mitochondria must maintain a balance between mitochondrial proliferation (biogenesis) and degradation (mitophagy). To mitigate degradation, mitochondria rely on intrinsic strategies to maintain quality. In this effort, Hur et al. [2] explored a novel role of HtrA2/OMI, a serine protease, in the regulation of mitochondrial homeostasis during hepatic fibrogenesis. The authors showed that overexpression of HtrA2/OMI led to antifibrotic effect due to CCl_4_, by enhancing the antioxidant activity of mitochondria in hepatocytes. 

In an unrelated study, but with a similar focus on mitochondria self-preservation during stress due to excess of Ca^2+^, Mishra et al. [3] reported an intriguing observation that cyclosporin A bolsters mitochondrial Ca^2+^ buffering capacity in a phosphate-dependent manner in guinea pig cardiomyocytes isolated mitochondria. This novel observation indicates that cyclosporin A activates, yet determined, mitochondrial molecular mechanisms involved in Ca^2+^ sequestration. This additional insight into the action of cyclosporin A could potentially reveal different therapeutic approaches targeted at regulating mitochondria Ca^2+^ homeostasis and reduce cardiac injury in Ca^2+^ overload.

*Mitochondrial and cellular metabolism*: Normal mitochondrial and cellular metabolisms are tightly coupled. In healthy conditions, mitochondria account for the majority of the ATP produced in the cell via OXPHOS. In healthy cardiomyocytes, most of the acetyl CoA consumed by the heart is from fatty acids, with the remainder from pyruvate. In their study, Toleikis et al. [4], investigated the effects of fatty acid oxidation-induced changes in mitochondrial morphology and conformational changes in adenine nucleotide translocase (ANT) on the kinetics of the regulation of mitochondrial respiration in rat skinned cardiac fibers. Fatty acids alone or in combination with pyruvic acid was the substrate. The key message from this study is that fatty acids could regulate cellular energy metabolism by increasing the affinity of the ADP/ATP transporter for ADP, via conformational changes of the transporter. This study provides new understandings of the metabolic changes in altered age-related cardiovascular diseases. 

In another study, Parodi-Rullán et al. [5] sought to elucidate whether ANT knockdown affects respiratory chain supercomplex formation in H9c2 cardiomyoblasts. This study is predicated on a previous observation by the same group that pharmacological inhibition of ANT disintegrated respirasome, the main respiratory chain supercomplex containing ETC complexes I, III, and IV, in cardiac mitochondria [6] suggesting an essential role of ANT in respirasome formation. ANT1 knockdown in the H9c2 cells reduced the ∆Ψ_m_ but increased total cellular ATP levels [5]. Furthermore, ANT1 downregulation did not alter the enzymatic activity of the ETC complexes I-IV but reduced the level of the respirasome. The results of this study not only confirm the previous observations of the role of ANT in respirasome formation, it also provides new convincing data, never reported previously with significant physiological implications for cellular metabolism. The reliance on electron transfer and the respirasome as key regulators of cellular metabolism was further reported by Ni et al. [7]. This study focused on the impact of specific mutations on two core subunits of complex I on metabolic reprogramming and disruption of electron transfer. The authors used an elaborate and integrative proteome and metabolome on human patient skin fibroblast (pluripotent cells). Mutations of the complex led to impaired integrity of the respirasome with increased ROS, increased NADH/NAD^+^ ratio, and a switch towards glycolysis for cellular metabolism. These observations link the intactness of the respiratory complexes to the ability of mitochondria to execute OXPHOS and preserve normal cellular metabolism. The switch to anaerobic metabolism and ROS production is emblematic of the disruption of mitochondrial metabolism. 

The prerequisite in bridging the gap between mitochondrial metabolism, thermal regulation, and body mass is mitochondrial uncoupling proteins (UCPs) and fatty acid oxidation. The physiological role of UCP3 in normal physiology, and its emerging role in pathophysiology, provide exciting potential for bridging this gap. Lombardi et al. [8] investigated the role of UCP3 in metabolic control in a situation where thermal stress was eliminated. There was no significant difference in weight gain and body composition between the two genotypes under low-fat diet; however, when animals were fed a high-fat diet, the UCP3 knockout animals showed enhanced energy efficiency and lean tissue mass. This novel observation indicates that temperature is the determinant factor for the outcome of metabolic effects elicited by UCP3.

Functional mitochondria are potentially key to tissue regeneration. Poženel et al. [9] looked at the potential contribution of mitochondrial metabolism in preserving the integrity of the human amniotic membrane (hAMs). The premise of this study is that in the common cell culture environment, the viability of amniotic cells decreases rapidly, but the underlying mechanisms of this phenomenon are unclear. Exposing hAM cells to tension or no tension, the study showed that tension applied to the cells in the culture environment displayed greater viability, in part, due to the preservation of mitochondrial bioenergetics and concomitantly reduced apoptotic events. These observations are a harbinger for improving stem cell maturation and tissue regeneration by using media conducive to mitochondrial preservation.

Cardiovascular diseases are associated with age and have a detrimental impact on the whole organism. Telomere length and telomerase activity play a role in cellular aging. In their review article Nalobin et al. [10] discussed the emerging role of telomere length and telomerase in cardiac development, aging, and regeneration. With the surge in interest in this topic and the contribution of mitochondria, the review is timely and highly appropriate. It postulates that the cumulative knowledge gained from the regenerative capacity of hearts will help in the formation of new approaches in the field of regenerative medicine for the treatment of diseases, for example, myocardial infarction and heart failure. 

Altering mitochondrial metabolism is the hallmark of numerous pharmacological agents with significant clinical utility. Herminghaus et al. [11] explored the untoward effects of two clinically effective drugs in the management of hypertriglyceridemia. Utilizing hepatic and colonic tissue homogenates of healthy rats, the study showed that both drugs negatively impacted hepatic mitochondrial metabolism, as manifested in diminished mitochondrial respiration and OXPHOS. In contrast, in colonic mitochondria, the drugs either did not significantly impact mitochondrial metabolism or increased it in some cases. This carefully executed study reveals that the side effects of these drugs are organ-specific. A note of caution is that the studies were conducted in the in vitro condition, and some of the dosages used are out of the clinical range. So, extrapolation to human experience is tempered. 

*Crosstalk between mitochondria and other subcellular compartments*: Mitochondria form an intricate network of connectivity with each other and with other cell structures, including the nucleus and the cytoskeleton. This dynamic interaction provides the framework for efficient mitochondrial function and cell survival. The anatomical and functional connection between mitochondria and the nucleus provides a coordinated cellular response to intracellular changes. The study by Eldarov et al. [12] explored the idea that mitochondria interaction with the nucleus is beyond intramembrane connection; it espouses the notion that a tiny fraction of the organelle could reside in the nucleus. This provocative concept has its genesis from earlier studies, but the results at the time generated skepticisms. Furthermore, other studies reported that mitochondria fragments found in the nucleus were indicative of pathology. With the advent of higher resolution technologies, this current study provided compelling new data obtained from healthy rat cardiomyocytes that support the localization of mitochondria in the nucleus. 

Two review articles discussed the crosstalk between mitochondria and the cytosolic compartment. A review contribution by Kuznetsov et al. [13] provided a detailed and insightful summary of the physiological relevance of the crosstalk between mitochondria and cytoskeletal proteins. The review highlights the role of these proteins on mitochondrial intracellular organization and interaction with other organelles, the regulation of mitochondrial function, ATP production, and energy transfer. This anatomical and functional coupling is the hub for the integration of mitochondrial function with normal cell physiology and the preservation of life. In a different perspective on the interaction between mitochondria and cytosolic constituents, Kotrys and Szczesny [14] reviewed the impact of the mitochondrial genome on normal cell physiology and pathophysiology. The mitochondrial genome encodes for only 13 proteins involved in mitochondrial respiration; however, they also encode RNAs, which influence cell physiology when released into the cytosol. The review specifically focuses on the latest knowledge on mitochondrial transcripts, including mitochondrial long non-coding RNAs and novel functions of these transcripts. These novel mitochondrial gene regulation transcripts extend the repertoire of potential mechanisms by which mitochondria influence cell physiology.

*Mitochondrial ion channels*: In mammals, mitochondrial K_Ca_ channels have been reported to regulate mitochondrial function and to provide protection against cell injury. A study by Gururaja Rao et al. [15] reported, for the first time, the presence of the BKCa (Slo) channel in mitochondria of the Drosophila (fruit fly). Mutation of the *slo* gene increased ROS generation, which could decrease survival and lifespan. The study is further bolstered by experiments that showed a reversal in mortality and improved lifespan following the overexpression of the human *slo* gene in the flies. The implications of the study are noteworthy; they provide new physiological understandings that may be relevant in our effort to decipher the underlying mechanisms of aging-related diseases.

## 3. Mitochondria in Diseases

Recently, different aspects of mitochondrial dysfunction have been associated with multiple human diseases, and hence, mitochondria are becoming a promising pharmacological target for the treatment of a broad range of diseases. This section of the Editorial comprising 11 articles focuses on the role of mitochondrial dysfunction in several pathological conditions. These studies can be grouped in the following four sections based on disease types: (1) *Neurological disorders*, (2) *Liver diseases,* (3) *Diseases associated with oxygen deficiency,* and (4) *Inborn and metabolic diseases.*

*Neurological disorders:* A study by Kim et al. [16] described a novel mechanism potentially regulating mitochondrial dynamics and seizure activity in the central nervous system. They provide novel evidence that transient receptor potential canonical channel-6 (TRPC6) regulates mitochondrial Lon protease 1 (LONP1) expression via the ERK1/2-mediated pathway. Activation of this pathway dramatically changes mitochondrial dynamics and assumed as an important therapeutic target for neuroprotection from various neurological diseases. In another study, the same group of authors [17] demonstrated that 2-cyano-3,12-dioxo-oleana-1,9(11)-dien-28-oic acid methyl ester (CDDO-Me), an analog of oleanolic acid exhibiting promising therapeutic effects in cancer, inflammatory, and neural diseases, irreversibly inhibits Lon protease-1 (LONP1) and activates ERK1/2 and c-Jun N-terminal kinase (JNK) pathways. They showed that CDDO-Me may selectively attenuate seizure activity in the cornu ammonis area 1 by rescuing the abnormal mitochondrial machinery, but in contrast to data reported above, this pathway was independent of LONP1 activity. Another report by Kho et al. [18] addressed the effect of glucose reperfusion after hypoglycemia on seizure, unconsciousness, and neuronal death. The data obtained by these authors suggest that abnormally elevated levels of pyruvate dehydrogenase kinase (PDK), and subsequent inhibition of pyruvate dehydrogenase play a critical role in this phenomenon. The authors found that sodium dichloroacetate, an inhibitor of PDK, can alleviate hippocampal neuronal death induced by hypoglycemia. 

*Liver diseases:* A study by Tan et al. [19] investigated the impact of lipid droplet accumulation on cellular oxidative stress. They have shown that overexpression of Perilipin 5 (PLIN5), a key lipid droplet protein required for the formation of contacts between mitochondria and lipid droplets, reduces ROS levels and improves mitochondrial function in HepG2 cells. They assume that the upregulation of PLIN5 is a survival strategy of cells in response to stress. Feichtinger et al. [20] examined cholangiocellular carcinoma biopsies in order to better understand the impact of mitochondria. They have found that the expression of voltage-dependent anion-selective channel 1 (VDAC-1) in the outer mitochondrial membrane inversely correlates with UICC (Union Internationale Contre le Cancer) cancer stage classification. Also, significantly lower survival was observed for low/moderate VDAC1 expressors compared to high expressors. These data suggest that lower mitochondrial mass is associated with shorter survival of patients with cholangiocellular carcinoma. Also, one review contributed to this section. Migliaccio at al. [21] contributed a review article summarizing recent findings on mitochondrial adaptive response and oxidative stress induction in the liver, the main tissue involved in fat metabolism and pollutant detoxification, and in male gonads, the main targets of endocrine disruption induced by both high-fat feeding and environmental pollutants. This review provided novel insights into the mechanisms underlying cellular response to the exposure to stressful environmental stimuli and metabolic adaptation to promote cellular survival. 

*Diseases associated with oxygen deficiency:* In a study by Graf et al. [22], they reported the changes in cerebellar amino acid metabolism in pregnancy with particular emphasis on the role of 2-oxoglutarate dehydrogenase complex. Hormonal changes occurring in pregnancy are known to coordinate a broad range of physiological adaptations, including changes in amino acid metabolism. The data obtained by this group suggest that these changes critically influence mitochondrial function and the resistance of pregnant rats to hypoxia. The authors suggest that specific patterns of amino acids and the activity of the α-ketoglutarate dehydrogenase complex in mitochondria can be used as sensitive markers for the adaptation to hypoxia. In a review article, Ferko et al. [23] summarized and discussed previous studies that evaluated the factors affecting the regulatory mechanisms in mitochondria at the level of mitochondrial permeability transition and its impact on comprehensive myocardial protection. The review put particular emphasis on signaling pathways leading to mitochondrial energy maintenance during partial oxygen deprivation. 

*Inborn and metabolic diseases:* Leber’s hereditary optic neuropathy (LHON), an inherited mitochondrial disease, was the focus of the study by Starikovskaja et al. [24]. The authors performed an entire mtDNA genome sequencing and provided genealogical and molecular genetic data on mutations and haplogroup background of LHON patients in Russia (Siberia) and Europe. The results indicate that haplogroup affiliation and the mutational spectrum of the Western Siberian LHON cohort substantially deviated from those of European populations. Another study by Riess et al. [25] was focused on the adverse effects of thiazolidinediones, a class of anti-diabetic drugs, which sometimes were associated with heart failure. The latter was not clear, because these drugs activate the peroxisome proliferator-activated receptor-gamma (PPARγ), which is believed to play a key role in cardioprotection. However, Riess and coauthors [25] showed that there is another PPARγ-independent mechanism of thiazolidinedione action based on a reversible increase in mitochondrial oxidation, causing an increase in ROS production and a decrease in membrane potential. Both mechanisms may cause damage to the myocardium and have to be considered in the treatment of diabetic patients. The study by Picca et al. [26] attempted to evaluate the impact of iron metabolism on the process of muscle aging with an emphasis on mitochondrial homeostasis. Their data show that the changes in iron metabolism are strongly associated with mtDNA content and damage. The authors assume that muscle iron homeostasis is altered in old age, which contributes to the loss of mtDNA stability and impairs muscle metabolism. Muscle iron metabolism may, therefore, represent a target for therapeutic interventions against muscle aging.

In conclusion, the Special Issue “Mitochondria in Health and Diseases” includes the most recent studies that elucidate the physiological role of mitochondria in cell life as well as the response of mitochondria to various pathological stimuli in cell/animal models of human diseases, and in patients. The articles in this Special Issue will further improve our understanding of mitochondrial biology under physiological and pathological conditions, and open new avenues for the development of new pharmacological compounds and conditional approaches for the treatment of human diseases through targeting mitochondria.
